# The predictive performance of current termination-of-resuscitation rules in patients following out-of-hospital cardiac arrest in Asian countries: A cross-sectional multicentre study

**DOI:** 10.1371/journal.pone.0270986

**Published:** 2022-08-10

**Authors:** Shu-Hsien Hsu, Jen-Tang Sun, Edward Pei-Chuan Huang, Tatsuya Nishiuchi, Kyoung Jun Song, Benjamin Leong, Nik Hisamuddin Nik AB Rahman, Pairoj Khruekarnchana, GY Naroo, Ming-Ju Hsieh, Shu-Hui Chang, Wen-Chu Chiang, Matthew Huei-Ming Ma

**Affiliations:** 1 Department of Emergency Medicine, National Taiwan University Hospital, Taipei, Taiwan; 2 Department of Emergency Medicine, National Taiwan University Hospital Hsin-Chu Branch, Hsin-Chu City, Taiwan; 3 Department of Emergency Medicine, Far Eastern Memorial Hospital, New Taipei City, Taiwan; 4 Faculty of Medicine, Department of Acute Medicine, Kindai University, Osaka, Japan; 5 Department of Emergency Medicine, College of Medicine, Seoul National University, Seoul, Republic of Korea; 6 Emergency Medicine Department, National University Hospital, Singapore, Singapore; 7 Department of Emergency Medicine, School of Medical Sciences, Health Campus, Universiti Sains Malaysia, Kota Bharu, Malaysia; 8 Department of Emergency Medicine, Rajavithi Hospital, Bangkok, Thailand; 9 Department of Health & Medical Services, ED-Trauma Centre, Rashid Hospital, Dubai, United Arab Emirates; 10 Institute of Epidemiology and Preventive Medicine, College of Public Health, Taipei, Taiwan; 11 Department of Emergency Medicine, National Taiwan University Hospital Yun-Lin Branch, Douliu City, Taiwan; Fondazione IRCCS Policlinico San Matteo, ITALY

## Abstract

**Background:**

Termination-of-resuscitation rules (TORRs) in out-of-hospital cardiac arrest (OHCA) patients have been applied in western countries; in Asia, two TORRs were developed and have not been externally validated widely. We aimed to externally validate the TORRs using the registry of Pan-Asian Resuscitation Outcomes Study (PAROS).

**Methods:**

PAROS enrolled 66,780 OHCA patients in seven Asian countries from 1 January 2009 to 31 December 2012. The American Heart Association-Basic Life Support and AHA-ALS (AHA-BLS), AHA-Advanced Life Support (AHA-ALS), Goto, and Shibahashi TORRs were selected. The diagnostic test characteristics and area under the receiver operating characteristic curve (AUC) were calculated. We further determined the most suitable TORR in Asia and analysed the variable differences between subgroups.

**Results:**

We included 55,064 patients in the final analysis. The sensitivity, specificity, negative predictive value, positive predictive value, and AUC, respectively, for AHA-BLS, AHA-ALS, Goto, Shibashi TORRs were 79.0%, 80.0%, 19.6%, 98.5%, and 0.80; 48.6%, 88.3%, 9.8%, 98.5%, and 0.60; 53.8%, 91.4%, 11.2%, 99.0%, and 0.73; and 35.0%, 94.2%, 8.4%, 99.0%, and 0.65. In countries using the Goto TORR with PPV<99%, OHCA patients were younger, had more males, a higher rate of shockable rhythm, witnessed collapse, pre-hospital defibrillation, and survival to discharge, compared with countries using the Goto TORR with PPV ≥99%.

**Conclusions:**

There was no single TORR fit for all Asian countries. The Goto TORR can be considered the most suitable; however, a high predictive performance with PPV ≥99% was not achieved in three countries using it (Korea, Malaysia, and Taiwan).

## Introduction

Termination-of-resuscitation rules (TORRs) provide a subjective decision concerning the balance of an effective resuscitation in patients with out-of-hospital cardiac arrest (OHCA) and the concept of medical futility. OHCA, with an annual incidence rate of 50–60 per 100,000 persons globally [1,2], is a high-maintenance medical condition with a low survival rate (5–20% worldwide and 2–11% in Asia) [1–3]. One Asian study using the Pan-Asian Resuscitation Outcomes Study (PAROS) registry revealed that OHCA survival rate is only 5.4% [4]. Since 1990, medical futility has been defined as imparting a <1% chance of survival after medical intervention [5–7]. To provide the greatest benefit to the population, medical re-allocation is required for an efficient workforce and use of bedspaces and medical devices, which are important during the COVID-19 pandemic [8–10]. Studies showed that the implementation of TORRs can reduce unnecessary transport [11], traffic accident [12,13], medical futility [14,15], and financial cost [16]. Various TORRs have been developed since 1980 due to different cultural, ethical, and legal demands under different time and space [13,17–21]. However, the validation of TORRs is as crucial as its development and should be prioritised before clinical implementation.

The performance of TORRs in western countries has been reviewed and well validated [22,23]. In Asia, TORRs are yet to be implemented, and the predictive performance remains controversial [20–21,24–26]. EMS systems are different from country to country; however, EMS systems in many Asian countries still have some fundamental similarities. EMS system is divided into Angio-American system (AAS) and Franco-German system (FGS). The EMS system in Asia is similar to the Angio-American system. For example, AAS EMS is responsible to deliver most of the patients to the emergency department (ED) for physician treatment, which is a concordance to Asia EMS; whereas in FGS, a physician is brought to the patients and very few patients approach the ED. Furthermore, as AAS, prehospital care is provided by paramedics in Asia; in FGS, prehospital care is provided by emergency physicians. Therefore, we selected TORRs developed in the United States and in Asia for analysis which is due to the consideration of different EMS systems [27]. Two TORRs, the AHA-BLS (American Heart Association- Basic Life Support) and AHA-ALS (American Heart Association- Advanced Life Support) TORRs, after being validated internally and externally for many years, are being clinically implemented in western countries and represent international guidelines recommended by the AHA in 2020 [11,19,28–33]. However, these TORRs lacked evidence of predictive performance in Asia. In Japanese studies, the AHA-BLS TORR showed good performance in mortality prediction [21,24,25]; however, several studies showed that the AHA-BLS TORR cannot achieve the definition of futile medicine in death prediction [20,26]. Asian studies also developed several TORRs; Goto and Shibahashi are both Asian TORR representatives. Goto et al. developed a TORR stating that physicians can cease resuscitation after arrival at the emergency department, which is consistent with most Asian legislation [20]. According to our knowledge, Malaysia is the only Asia country that legislation is able to terminate CPR in the field. Shibahashi et al. proposed a TORR that provides objective criteria in quick decision-making after the emergency medical service (EMS) personnel arrive at the scene, which involves pre-hospital termination and is favourable in the future while EMS personnel in Asia can legally perform termination-of-resuscitation (TOR) in the field [21]. However, the above two TORRs are only validated in Japan and were not yet assessed in other Asian countries.

In this study, four TORRs were selected for performance evaluation: the AHA-BLS, AHA-ALS, Goto, and Shibahashi TORRs. We aimed to compare the predictive performance of selected TORRs using the data obtained from the PAROS registry, to find out the most suitable TORR in Asia and to explore the potential relation of suboptimal performance of TORRs.

## Materials and methods

### Study design and setting

We used the PAROS retrospective multinational cohort to validate the performance of TORRs in OHCA patients in 7 Asian countries in Asia, and then selected a suitable TORR for most Asian countries. We also further classified these 7 countries into two groups: countries with good TORR performance and countries with suboptimal TORR performance based on the PPV value of the chosen TORR. This is to explore the differences between the two groups.

The PAROS study established a registry for multinational participants to collect 1.5–2.5-year OHCA data from 1 January 2009 to 31 December 2012 [3]. First, variables were derived based on Utstein registry [34], under a standardized record template. Second, the extracted information (derived from dispatch, ambulance, and emergency department records) was imported into an online data registry called ePAROS. To ensure data precision, designated coordinators in each country affirmed the collected data before and after data entry in the ePAROS system; the PAROS study coordinating centre further verified and clarified the data completeness, data range, and logical consistency with the corresponding person in each country through data-source verification.

This study was approved by the Institutional Review Board of the National Taiwan University Hospital (NTUH). The PAROS study received national institutional review boards (IRBs) approval from the local committees of all participating countries and all IRB approvals were maintained in the coordination centre [3]. Patient consent was waived due to de-identified PAROS registry dataset. PAROS Clinical Research Network use patient’s names or identification numbers to match the EMS records with hospital outcomes. After the record is completed, patients’ identifiers were removed from the dataset by PAROS personnel to safeguard the privacy of patients. Moreover, there is a data sharing agreement of PAROS registry to protect the confidentiality of patients enrolled in the study.

### Study population

The study population included adults, non-traumatic OHCA patients between 1 January 2009 and 31 December 2012, identified from the PAROS registry. The PAROS data encompass twelve communities from seven countries including Japan, Malaysia, Singapore, South Korea, Taiwan, Thailand, and the United Arab Emirates (Dubai) [3]. The exclusion criteria include: (1) age <18 years, (2) non-EMS transport to the emergency department, (3) traumatic cardiac arrest OHCA patients, (4) obvious signs of death (e.g. decapitation, rigor mortis, lividity, and decapitation) or having do-not-resuscitate (DNR) orders, and (5) missing data despite meeting the inclusion criteria.

### Variables

The variables were based on the four selected TORRs: the AHA-BLS, AHA-ALS, Goto, and Shibahashi TORRs. Patient demographics, prehospital information with definition followed by Utstein recommendation [35], and survival status were extracted including age, sex, prehospital rhythm, prehospital defibrillation, bystander cardiopulmonary resuscitation (CPR), cardiac arrest witnessed, prehospital return of spontaneous circulation ROSC and survival to discharge. The prehospital ROSC is prehospital status, which means ROSC status before emergency department arrival; whereas survival to discharge is the overall survival of OHCA patients after hospital discharge.

In most PAROS countries, “survival to hospital discharge” was a necessary information in the registry, while in Japan the item was substituted by "survival to 30-day hospitalisation”. Both of these were acceptable to the Utstein registry template [34]. Therefore, “survival to hospital discharge” in this study had two definitions.

### Outcome measures

The prediction outcome is in-hospital mortality. The tests of the TORRs for the prediction of in-hospital mortality are evaluated by sensitivity (Sn), specificity (Sp), positive predictive values (PPV), negative predictive value (NPV), and area under the receiver operating characteristic curve (AUC). Medical futility is defined as medical managements that provide ≤1% chance of survival [5,36]. Countries with PPV ≥99% were considered as having good TORR performance, whereas countries with PPV <99% were considered as having suboptimal TORR performance.

### Statistical analysis

Means and standard deviations (SD) were reported for continuous variables. Counts and percentages were calculated for categorical variables. The overall performance of the four TORRs were summarised by determining the Sn, Sp, PPV, NPV with 95% CI for mortality prediction outcome, and AUC. The comparison of the variable differences between subgroups was examined using the Student t-test and chi-square test, as appropriate. Statistical significance was defined as P<0.05. All statistical analyses were calculated using SAS software version 9.4 (SAS Institute, Cary, NC, USA).

## Results

**Fig 1:**
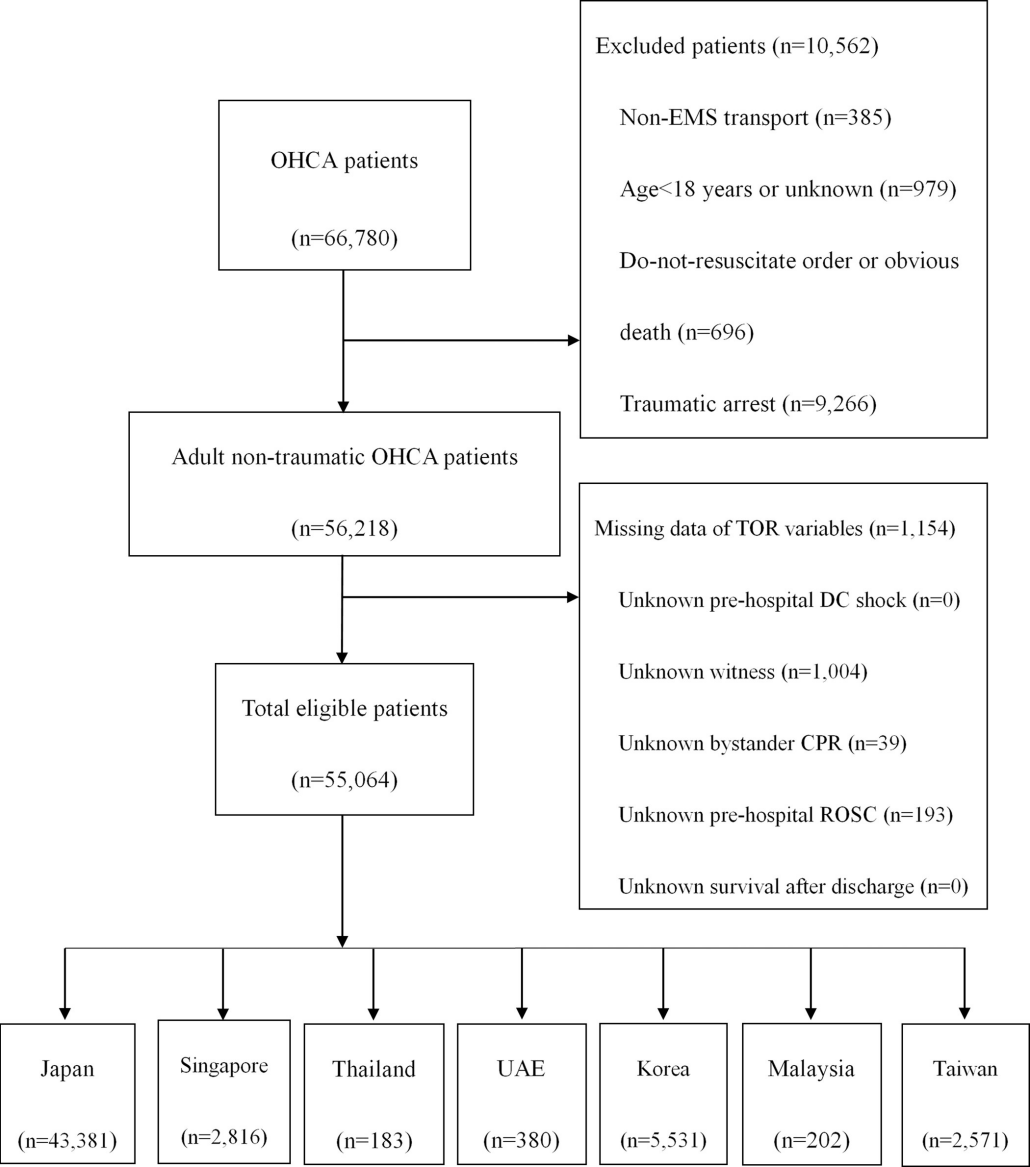
Patient flow chart.

From 1 January 2009 to 31 December 2012, 66,780 patients were enrolled in the PAROS registry. In total, 11,716 patients were excluded and, finally, 55,064 patients were reviewed; they were further classified into subgroups according to different countries. Most OHCA patients were from Japan, and the patient flow chart is shown in Fig 1.

Table 1 shows the demographic information of the 55,064 individuals. The mean±SD age of the overall population was 76.00±15.8 years; 58.5% of patients (n = 32,185) were >73 years old, and 59.5% of patients were males (n = 32,750). In total, 57.0% of the cases were not witnessed, 35.6% were witnessed by a bystander, and 7.4% were witnessed by an emergency medical technician (EMT). In total, 37.3% of the patients received pre-hospital bystander CPR and 14.0% received pre-hospital defibrillation. Most of the patients (88.2%) showed non-shockable rhythm, 0.3% showed ventricular tachycardia, and 7.8% showed ventricular fibrillation. Only a few patients (8.5%) achieved pre-hospital ROSC, and 6.0% survived to hospital discharge or survived beyond 30 days during hospitalisation.

**Table 1:** Patient characteristics.

	Overall (n = 55,064)	Japan (n = 43,381)	Singapore (n = 2,816)	Thailand (n = 183)	UAE (n = 380)	Korea (n = 5,531)	Malaysia (n = 202)	Taiwan (n = 2,571)
**Age, year (mean±SD)**	76.0 (15.8)	74.6 (15.0)	65.5 (15.6)	60.7 (19.1)	51.7 (16.1)	68.0 (16.4)	59.0 (16.4)	77.0 (16.4)
**Age ≥73 y/o^a^, n (%)**	32,185 (58.5)	27,333 (61.6)	1,011 (35.9)	61 (33.3)	44 (11.6)	2,181 (39.4)	42 (20.8)	1,513 (58.9)
**Male, n (%)**	32,750 (59.5)	25,036 (56.4)	1,864 (66.2)	121 (66.1)	315 (82.9)	3,633 (65.7)	141 (69.8)	1,640 (63.8)
**Shockable rhythm**	5,646 (10.2)	3,733 (8.6)	542 (19.2)	14 (7.7)	79 (20.8)	1,014 (18.3)	8 (4.0)	256 (10.0)
**VT**	177 (0.3)	74 (0.2)	16 (0.5)	0 (0.0)	6 (1.6)	74 (1.3)	0 (0.0)	7 (0.3)
**VF**	4,299 (7.8)	2,615 (6.0)	514 (18.3)	14 (7.7)	67 (17.6)	839 (15.2)	2 (1.0)	248 (9.6)
**Unknown shockable**	1,170 (2.1)	1,044 (2.4)	12 (0.4)	0 (0.0)	6 (1.6)	101 (1.8)	6 (3.0)	1 (0.04)
**Non-shockable rhythm**	48,560 (88.2)	39,612 (91.3)	2,272 (80.7)	111 (60.6)	301 (79.2)	4,125 (74.6)	128 (63.4)	2,011 (78.2)
**Asystole**	27,335 (49.6)	20,695 (47.7)	1,505 (53.4)	93 (50.8)	261 (68.7)	3,202 (57.9)	50 (24.8)	1,529 (59.5)
**PEA**	8,243 (15.0)	6,219 (14.3)	748 (26.6)	17 (9.3)	34 (8.9)	758 (13.7)	3 (1.5)	464 (18.0)
**Unknown non-shockable**	12,982 (23.6)	12,698 (29.3)	19 (0.7)	1 (0.5)	6 (1.6)	165 (3.0)	75 (37.1)	18 (0.7)
**Unknown**	858 (1.6)	36 (0.1)	2 (0.1)	58 (31.7)	0 (0.0)	392 (7.1)	66 (32.6)	304 (11.8)
**Pre-hospital defibrillation, n (%)**	7,695 (14.0)	5,080 (11.4)	682 (24.2)	27 (14.8)	140 (36.8)	1,449 (26.2)	9 (4.5)	308 (12.0)
**Cardiac arrest witnessed, n (%)**								
**Not witnessed**	31,398 (57.0)	25,609 (59.0)	1,211 (43.0)	52 (28.4)	192 (50.5)	2,508 (45.3)	72 (35.6)	1,754 (68.2)
**Bystander-witnessed**	19,602 (35.6)	14,588 (33.6)	1,399 (49.7)	117 (63.9)	176 (46.3)	2654 (48.0)	108 (53.5)	560 (21.8)
**EMS-witnessed**	4,064 (7.4)	3,184 (7.4)	206 (7.3)	14 (7.7)	12 (3.2)	369 (6.7)	22 (10.9)	257 (10.0)
**Bystander CPR, n (%)**	20,521 (37.3)	16,716 (38.5)	635 (22.6)	38 (20.8)	39 (10.3)	2328 (42.1)	61 (30.2)	704 (27.4)
**Pre-hospital ROSC, n (%)**	4,653 (8.5)	3,831 (8.8)	145 (5.2)	39 (21.3)	14 (3.7)	307 (5.6)	7 (3.5)	310 (12.1)
**Survival to discharge**	3,287 (6.0)	2,390 (5.5)	89 (3.2)	11 (6.0)	13 (3.4)	591 (10.7)	5 (2.5)	188 (7.3)

^a^Age ≥73 y/o is one of the TOR criteria in the Shibahashi TORR.

VT, ventricular tachycardia; VF, ventricular fibrillation; PEA, pulseless electrical activity; EMS, emergency medical service; CPR, cardiopulmonary resuscitation; ROSC, return of spontaneous circulation.

Table 2 shows the performance of the four TORRs according to the countries. Countries with good Goto TORR performance included Japan, Singapore, Thailand, and UAE, and overall data; for AHA-BLS TORR, Singapore, and UAE; for AHA-ALS TORR, Japan, Singapore, and UAE; and for Shibahashi TORR, Japan, Singapore, Thailand, UAE, and overall data.

**Table 2:** Performance of the 4 TORRs to predict in-hospital mortality.

	Goto				AHA-BLS				AHA-ALS				Shibahashi			
	Sn^a^	Sp^b^	PPV^c^	NPV^d^	Sn	Sp	PPV	NPV	Sn	Sp	PPV	NPV	Sn	Sp	PPV	NPV
**Overall** **95%CI**	53.8 53.3–54.2	91.4 90.4–92.4	99.0 98.9–99.1	11.2 10.8–11.5	79.0 78.6–79.4	80.0 79.5–82.2	98.5 97.5–99.6	19.6 19.0–20.3	48.6 48.2–49.0	88.3 87.2–89.4	98.5 98.3–98.6	9.8 9.5–10.2	35.0 34.6–35.4	94.2 93.4–95.0	99.0 98.8–99.1	8.4 8.1–8.7
**Japan** **95%CI**	56.9 56.4–57.4	94.0 93.0–94.9	99.4 99.3–99.5	11.3 10.8–11.7	81.1 80.7–81.4	85.7 84.3–87.1	98.9 98.9–99.1	20.9 20.1–21.7	48.3 47.8–48.8	91.6 90.5–92.7	99.0 98.9–99.1	9.4 9.0–9.7	38.9 38.5–39.4	94.1 93.2–95.0	99.1 99.0–99.3	8.3 7.9–8.6
**Singapore** **95%CI **	34.8 33.0–36.6	97.8 94.7–100	99.8 99.5–100	4.7 3.7–5.6	69.6 67.9–71.3	93.3 88.0–98.5	99.7 99.4–99.9	9.1 7.2–11.0	55.0 53.2–56.9	95.5 91.2–99.8	99.7 99.5–99.9	6.5 5.2–7.8	14.2 12.8–15.5	98.9 96.7–100	99.7 99.2–100	3.6 2.9–4.4
**Thailand** **95%CI**	20.9 14.9–27.0	100.0 100 -100	100.0 100 -100	7.5 3.2–11.7	66.9 59.8–73.9	81.8 59.0–100	98.3 95.9–100	13.6 5.4–21.9	54.1 46.6–61.5	81.8 59.0–100	97.9 95.0–100	10.2 3.9–16.6	10.5 5.9–15.0	100.0 100 -100	100.0 100 -100	6.7 2.9–10.5
**UAE** **95%CI**	35.2 30.0–40.0	92.3 77.8–100	99.2 97.7–100	4.8 2.2–7.5	61.9 56.9–66.8	84.6 65.0–100	99.1 97.9–100	7.3 3.1–11.4	56.1 51.1–61.2	92.3 77.8–100	99.5 98.6–100	6.9 3.2–10.7	6.5 4.0–9.1	100.0 100 -100	100.0 100 -100	3.7 1.7–5.6
**Korea** **95%CI**	38.6 37.3–40.0	83.8 80.8–86.7	95.2 94.3–96.1	14.0 12.9–15.2	70.9 69.6–72.2	63.6 59.7–67.5	94.2 93.5–95.0	20.7 18.9–22.6	40.8 39.4–42.1	78.2 74.8–81.5	94.0 93.0–95.0	13.6 12.5–14.8	15.2 14.2–16.2	96.1 94.5–97.7	97.0 95.8–98.2	11.9 11.0–12.9
**Malaysia** **95%CI**	33.5 26.9–40.1	80.0 44.9–100	98.5 95.6–100	3.0 0.0–6.0	82.2 76.9–87.6	40.0 0.0–82.9	98.2 96.1–100	5.4 0–13.0	56.3 49.4–63.3	60.0 17.1–100	98.2 95.8–100	3.4 0.0–7.0	6.09 2.8–9.4	80.0 44.9–100	92.3 77.8–100	2.1 0.0–4.0
**Taiwan** **95%CI**	59.9 58.0–61.9	79.3 73.5–85.1	97.3 96.5–98.2	13.5 11.5–15.5	74.1 72.4–75.9	67.6 60.9–74.2	96.7 95.8–97.5	17.1 14.4–19.8	60.1 58.1–62.0	75.5 69.4–81.7	96.9 96.0–97.8	13.0 11.0–15.0	40.6 38,6–42.6	86.2 81.2–91.1	97.4 96.4–98.4	10.3 8.8–11.8

^a^Sn, sensitivity: Number of dead individuals that TORR suggested termination of resuscitation divided by the number of total dead individuals following OHCAs.

^b^Sp, specificity: Number of individuals who survived that TORR suggested transportation divided by the total individuals who survived following OHCAs.

^c^PPV, positive predictive values: Number of dead individuals that TORR suggested termination of resuscitation divided by the total individuals that TORR suggested termination of resuscitation.

^d^NPV, negative predictive values: Number of individuals who survived that TORR suggested transportation divided by the total individuals that TORR suggested transportation.

AHA-BLS, American Heart Association Basic Life Support; AHA-ALS, American Heart Association Advanced Life Support.

Table 3 summarizes the four TORRs being used in Asia, including AUC, criteria, advantages, and disadvantages. The AHA-BLS TORR had the highest overall AUC with two countries showing good performance. The AHA-ALS TORR had the lowest AUC with three countries showing good performance. AHA-BLS and AHA-ALS TORRs can reduce transport rate but are not yet legally authorised in Asian countries. The Shibahashi TORR revealed good prediction performance in four Asian countries, and due to the objective criteria, the Shibahashi TORR was able to reduce transport rate by declaring death upon EMT arrival; however, the Shibahashi TORR still raises legal concern in Asia. The Goto TORR revealed good performance in 4 Asian countries and had the second highest AUC. In Goto TORR, death was declared by a physician upon arrival in the emergency department, which cannot reduce transport rate but is legally authorised in Asia.

**Table 3:** Summarized application of variant TORRs in Asia.

	Goto	AHA-BLS	AHA-ALS	Shibahashi
**Overall AUC**	0.73 (0.72–0.73)	0.80 (0.79–0.81)	0.60 (0.58–0.61)	0.65 (0.64–0.65)
**Countries with good performance^a^**	4 (Japan, Singapore, Thailand, UAE)	2 (Singapore, UAE)	3 (Japan, Singapore, UAE)	4 (Japan, Singapore, Thailand, UAE)
**Criteria**	1. Unshockable initial rhythm 2. Unwitnessed by bystanders 3. No prehospital ROSC	1. No pre-hospital shock delivered 2. Unwitnessed by EMT 3. No pre-hospital ROSC	1. No pre-hospital shock delivered 2. Unwitnessed by EMT or bystanders 3. No pre-hospital ROSC 4. No bystander CPR	1. Unshockable initial rhythm 2. Unwitnessed by bystanders 3. Age ≥73 years
**Advantage in utility in Asia**	1. Death declaration in ED by physician 2. Legally authorised	1. Reduced transport rate	1. Reduced transport rate	1. Death declaration immediately after the arrival of EMT 2. Reduced transport rate
**Disadvantage in utility in Asia**	1. Need transport to ED, cannot reduce transport time	1. Death declaration by EMT at scene 2. Not legally authorised	1. Death declaration by EMT at scene 2. Not legally authorised	1. Death declaration by EMT at scene 2. Not legally authorised

^a^Good performance: A PPV ≥99% in prediction of in-hospital mortality.

AHA-BLS, American Heart Association Basic Life Support; AHA-ALS, American Heart Association Advanced Life Support; AUC, area under the receiver operating characteristic curve; EMT, emergency medical technicians; ED, emergency department; CPR, cardiopulmonary resuscitation; ROSC, return of spontaneous circulation.

Table 4 shows that, compared with their counterpart, in the suboptimal Goto TORR performance group, patients were younger, had a higher percentage of males, a higher rate of shockable rhythm, witnessed collapse, pre-hospital defibrillation, and survival to discharge, but had a lower rate of pre-hospital ROSC. We selected Goto TORR as the suitable TORR in most Asian countries included in the study. Four Asian countries showed good Goto TORR performance. In Goto TORR, patients were sent to the hospital and death was declared by doctors, which is concordant with most Asian legislation and culture. To clarify the differences between countries with good and suboptimal performance, we categorised the seven countries using the Goto TORR into two groups according to the PPV value: the good TORR performance group (Japan, Singapore, Thailand, and UAE with PPV ≥99%) and the suboptimal TORR performance group (Korea, Malaysia, and Taiwan, with PPV <99%). Patient characteristics showed statistically significant differences when comparing the variables between the two groups.

**Table 4:** Patient characteristics in countries with good Goto TORR performance vs. suboptimal Goto TORR performance.

	Good Goto TORR performance^a^ (n = 46,760)	Suboptimal Goto TORR performance^b^ (n = 8,304)	P
**Age, year (mean, SD)**	73.8 (15.4)	67.6 (16.8)	<0.0001
**Gender, Male, n (%)**	27,336 (58.5)	5,414 (65.2)	<0.0001
**Type of initial rhythm, n (%)**			<0.0001
**Shockable**	4,368 (9.3)	1,278 (15.4)	
**VF**	3,210 (6.9)	1,089 (13.1)	
**VT**	96 (0.2)	81 (0.98)	
**Unknown shockable**	1,062 (2.3)	108 (1.3)	
**Non-shockable**	42,296 (90.5)	6,264 (75.4)	
**Asystole**	22,554 (48.2)	4,781 (57.6)	
**PEA**	7,018 (15.0)	1,225 (14.8)	
**Unknown non-shockable**	12,724 (27.2)	258 (3.1)	
**Unknown**	96 (0.2)	762 (9.2)	
**Pre-hospital defibrillation, n (%)**	5,929 (12.7)	1,766 (21.3)	<0.0001
**Cardiac arrest witness, n (%)**			<0.0001
**No witness**	27,064 (57.9)	4,334 (52.2)	
**EMS**	3,416 (7.3)	648 (7.8)	
**Bystander**	16,280 (34.8)	3,322 (40.0)	
**Bystander CPR, n (%)**	17,428 (37.3)	3,093 (37.3)	0.97
**Pre-hospital ROSC, n (%)**	4,029 (8.6)	624 (7.5)	0.0009
**Survival to discharge, n (%)**	2,503 (5.4)	784 (9.4)	<0.0001

^a^Good Goto TORR performance: Country with PPV≧99% belongs to this category, including Japan, Singapore, Thailand, UAE.

^b^Suboptimal Goto TORR performance: Country with PPV<99% belongs to this category, including Korea, Malaysia, Taiwan.

TORR, termination-of-resuscitation rule; VT, ventricular tachycardia; VF, ventricular fibrillation; PEA, pulseless electrical activity; EMS, emergency medical service; CPR, cardiopulmonary resuscitation; ROSC, return of spontaneous circulation.

## Discussion

### Main findings

There were three major findings in this study. First, there was no universal TORR that could be perfectly applied in all Asian countries; none of the four TORRs achieved a PPV ≥99% in prediction of death in Korea, Malaysia, and Taiwan. Second, under the circumstance, the Goto TORR was the suitable for most Asian countries included in this study by the high predictive performance and legislation requirement. The Shibahashi TORR may be favoured if the EMS has the authority to decline resuscitation at the scene. Third, TORR performance would be probably affected in countries with younger or with a higher male population. These findings are beneficial to the knowledge gap in the TORR applications of the current resuscitation guidelines.

### Interpretation of the study

In previous studies, the performance of TORRs in Asian countries remains controversial. Our study revealed that no universal TORR can be applied in pan-Asia. None of the four TORRs achieved a PPV ≥99% in survival prediction in Korea, Malaysia, and Taiwan. Under different population characteristics, EMS, and medical system, this study showed diversity in TORR performance among Asian countries. In Malaysia, an ambulance is staffed with medical assistance and the highest pre-hospital service level is a physician; these factors may have positive influence on the outcome of OHCA patients [4,37]. Several studies showed that, with the presence of a pre-hospital physician, OHCA patients showed better survival outcome and favourable neurological outcome [38–40]. Online Supplement Table 2 showed the analysis of prehospital time in seven Asia countries. We defined the prehospital time as from call to ambulance arrived ED. The two countries with the shortest prehospital time are Korean and Taiwan. Online Supplement Table 3 showed univariate analysis between good Goto TORR performance group and suboptimal performance group. In univariate analysis between good Goto TORR performance and suboptimal performance group, prehospital time is longer in good Goto TORR performance with statistics significant. In Korea and Taiwan, OHCA patients had relatively short prehospital resuscitation time and prolonged overall resuscitation time (pre-hospital EMT resuscitation and hospital resuscitation). Our study reported a relatively short prehospital time in Taiwan and Korea which is concordance with previous study [37]. One PAROS study revealed that the time from EMT arrival to hospital arrival is less than 20 minutes in Korea (13.0 minutes) and in Taiwan (17.0 minutes) [4]. The 2020 AHA guideline and study revealed that a universal TORR showed good performance in futility prediction at >20 minutes of CPR [41,42]. One study reported that 90% of OHCA patients regained pulse after 20 minutes of resuscitation and 99% of patients obtained pulse after 37 minutes of resuscitation [41]. In Asia, every OHCA patient will be sent to the hospital, thereby resulting in prolonged resuscitation time of >37 minutes. With pre-hospital resuscitation time <20 minutes and extended post-hospital resuscitation time, TORRs in Korea and Taiwan may fail to achieve medical futility. ROSC is related to resuscitation time; therefore, the timing of TORR assessment may contribute to the performance of TORR. In countries with short pre-hospital resuscitation time, TORR may also be different compared to countries with long pre-hospital resuscitation time. Although prehospital time may influence the predictive performance of TORRs rules, in ethical aspect, we believe prehospital time should not be one of the criteria of TORR unless in extremely remote regions. Further study will be required to determine an optimal TOR strategy and to figure out the proper assessment time in countries with short prehospital time; resuscitation time scale should also be considered in choosing the suitable TORR, including pre-hospital resuscitation and hospital resuscitation, in the future.

Online Supplement Table 1 showed the analysis of ED ROSC rate and the analysis of mechanical CPR including prehospital use and in-hospital use (at ED). In this study, prehospital mechanical CPR accounted for a very low proportion of OHCA patients; only Singapore has 248 cases with prehospital mechanical CPR recorded. Online Supplement Table 3 showed univariate analysis between good Goto TORR performance group and suboptimal performance group. In univariate analysis, prehospital mechanical CPR showed statistics significant between 2 groups; however, Singapore is the only country contributing the case number for prehospital mechanical CPR which could not represent the general condition of good Goto performance group. This study did not include ED status and in-hospital treatment for OHCA patients; thus, in the information of in-hospital use of mechanical CPR and ED ROSC, a large proportion of missing data made further analysis difficult.

### Application of the findings

By using the Goto and Shibahashi TORRs, more Asian countries achieved the definition of medical futility, including Japan, Singapore, Thailand, and UAE. The Shibahashi TORR led to a reduction in transport rate, immediate termination of resuscitation at scene by an EMT, and all the variables were objectively evaluated. However, considering that the EMTs in Asia are still not authorised to declare death at scene, applying the Shibahashi TORR may have legal and ethical concerns. Thus, the Shibahashi TORR may be more suitable in Asia once they have the authority to declare death at scene in the future. In the Goto TORR, futile resuscitation can be terminated after transport to the emergency department, and it is performed by legally authorised doctors. Using the Goto TORR cannot relieve the burden of the EMTs by reducing transport rate since the termination of resuscitation is performed in the hospital. However, the implementation of the Goto TORR can relieve the burden in the emergency department, and it is legally mandated in Asia. Considering the recent legality and ethical requirement in Asia, the Goto TORR is suitable in planning for a TORR policy.

### Factors associated with TORRs performance

In this study, we found that PPV and specificity were lower in three countries with Goto TORR performance, which may indicate that more OHCA patients which are eligible for the Goto TORR survived in these three countries, i.e. specificity values in Korea, Malaysia, and Taiwan were 38.6%, 33.5%, and 59.9%, respectively, which means that 61.4%, 66.5%, and 40.1% survivors, respectively, were eligible for the Goto TORR. In seven Asian countries, OHCA patients are sent to hospital; however, in Korea, Malaysia, and Taiwan, specificity was lower compared to the other four countries. The mean age in the suboptimal group was 68.6 years and that in the good performance group was 73.8 years old, which is in concordance with the Shibahashi TORR. In the Shibahashi study, one of the TOR criteria was age <73 years; OHCA patients aged <73 years were transported [21]. A PAROS study showed a higher rate of pre-hospital ROSC in younger patient and males [43]. Another PAROS study reported that the males had a higher rate of pre-hospital ROSC and survival to discharge [44]. TORR performance may be diminished since patients with a younger age or males had a higher probability of regaining pulse, thereby requiring hospital transport. These patients may benefit from prolonged resuscitation or advance medical treatment [41]. However, the age cut-off point and gender issue are under an ethical debate, and further evaluation will be required.

### Limitations

There are several limitations in this study. First, we conducted the study by using PAROS registry which is a city-based registry without national coverage; therefore, the results are unable to undermine the influence of selection bias and generalize to the entire Asia population. Second, the sample size was unevenly distributed among the countries and, therefore, small in some countries, resulting in unstable test performance. We reviewed 55,064 OHCA cases to conduct an external validation of each TORR in Asia. However, to preserve the diversity among countries, we conducted individual analysis on each country. Third, 1,154 patients with missing data were excluded from 56,218 OHCA patients. However, the 2.1% missing data rate is reasonable considering the huge database. Fourth, the data we used were collected from 2009 to 2012. We know the data is not very updated because it contains the phase one data of the PAROS registry. However, our study focuses on the predictive performance of TORRs across Asian countries and provides a continental-wide understanding of TORRs on a pan-Asia scale. Therefore, with the influence of widely implemented strategies for BLS promotion and advanced life supports in EMS system [45–47], we believe this study is still informative to the readers in the prehospital resuscitation science. Fifth, the PAROS registry provided data on all outcomes of OHCA patients after medical treatment, but the hospital variables were not recorded; this study lacked the information regarding hospital care. TORRs are rules for prehospital decisions, to decide whether OHCA patients need transfer to the hospital or should terminate resuscitation in the field. Due to TORRs being the rules for prehospital settings, the criteria included in universal TORRs are all prehospital variables; in-hospital management of the patients usually is not included as one of TORRs criteria. However, the rapid evolution of medical technology may affect the survival rate of OHCA patients; therefore, the applicability of TORRs should be examined regularly.

## Conclusions

There is no single TORR fit for all Asian countries included in the study. The Goto TORR was considered the most suitable in Asia based on its predictive performance and content. In countries using the Goto TORR with PPV<99% (Korea, Malaysia, and Taiwan), the OHCA population was younger, comprised more males, had higher rates of shockable rhythm, witnessed collapse, pre-hospital defibrillation, and survival to discharge.

## Supporting information

S1 File (DOCX)

## Abbreviations

TORR: termination-of-resuscitation rule
OHCA: out-of-hospital cardiac arrest
PAROS: Pan-Asian Resuscitation Outcomes Study
AHA-BLS: American Heart Association Basic Life Support
AHA-ALS: American Heart Association Advanced Life Support
Sn: sensitivity
Sp: specificity
PPV: positive predictive value
NPV: negative predictive value
AUC: area under the receiver operating characteristic curve
DNR: do-not-resuscitate
EMS: emergency medical services
EMT: emergency medical technicians
ROSC: return of spontaneous circulation
CPR: cardiopulmonary resuscitation
