# Supplementary material for: The predictive performance of current termination-of-resuscitation rules in patients following out-of-hospital cardiac arrest in Asian countries: A cross-sectional multicentre study
Source: PLoS One. 2022 Aug 10;17(8):e0270986. doi: 10.1371/journal.pone.0270986 (PMC9365191; doi:10.1371/journal.pone.0270986)
Supplement: S1 File — (DOCX) [file pone.0270986.s001.docx]

**Online Supplement Table 1** Patient characteristics in 7 Asia countries.

|  | **Overall (n=55,064)** | **Japan (n=43,381)** | **Singapore (n=2,816)** | **Thailand (n=183)** | **UAE (n=380)** | **Korea (n=5,531)** | **Malaysia (n=202)** | **Taiwan (n=2,571)** |
| --- | --- | --- | --- | --- | --- | --- | --- | --- |
| **ED ROSC, n (%)** | | | | | | | | |
| **ROSC, n (%)** | 7883(14.3) | 4182(9.6) | 736(26.1) | 51(27.9) | 22 (5.8) | 2088(37.8) | 17(8.4) | 787(30.6) |
| **No ROSC** | 14876(27.0) | 8236(19.0) | 1997(70.9) | 51(27.9) | 347 (91.3) | 2458(44.4) | 80(39.6) | 1707(66.4) |
| **Missing data** | 32305(58.7) | 30963(71.4) | 83(3.0) | 81(44.3) | 11(2.9) | 985(17.8) | 105(52.0) | 77(3.0) |
| **Prehospital Mechanical CPR, n (%)** | | | |  |  |  |  |  |
| **Prehospital Mechanical CPR use, n (%)** | 248(0.5) | 0 | 248(8.8) | 0 | 0 | 0 | 0 | 0 |
| **No Prehospital Mechanical CPR use, n (%)** | 54808(99.5) | 43381(100) | 2568(91.2) | 183(100.0) | 380(100.0) | 5531(100.0) | 194(96.0) | 2571(100.0) |
| **Missing data** | 8(0.0) | 0 | 0 | 0 | 0 | 0 | 8(4.0) | 0 |
| **ED Mechanical CPR, n (%)** | | |  |  |  |  |  |  |
| **ED Mechanical CPR use, n (%)** | 1017(1.9) | 0 | 674(23.9) | 0 | 23(6.0) | 318(5.7) | 2(1.0) | 0 |
| **No ED Mechanical CPR use, n (%)** | 2713(4.9) | 0 | 2142(76.1) | 117(63.9) | 357(94.0) | 5213(94.3) | 97(48.0) | 0 |
| **Missing data** | 51334(93.2) | 43381(100) | 0 | 66(36.1) | 0 | 0 | 103(51.0) | 2571(100.0) |

**ED, emergency department; ROSC, return of spontaneous circulation; CPR, cardiopulmonary resuscitation**

**Online Supplement Table 2** Call to ED time in 7 Asia countries.

|  | **Overall (n=55,064)** | **Japan (n=43,381)** | **Singapore (n=2,816)** | **Thailand (n=183)** | **UAE (n=380)** | **Korea (n=5,531)** | **Malaysia (n=202)** | **Taiwan (n=2,571)** |
| --- | --- | --- | --- | --- | --- | --- | --- | --- |
| **Call to ED time, mean (min)** | 41.3 (2244.1) | 45.3 (2523.9) | 34.0  (7.9) | 45.6  (22.7) | 35.6  (11.3) | 21.5  (7.9) | 42.6  (22.0) | 24.3  (8.5) |

**ED, emergency department**

**Online Supplement Table 3** Patient characteristics in countries with good Goto TORR performance vs. suboptimal Goto TORR performance

|  | **Good Goto TORR performance* (n=46,760)** | **Suboptimal Goto TORR performance†(n=8,304)** | **P** | **Missing data** |
| --- | --- | --- | --- | --- |
| **ED ROSC, n (%)** | 4991(10.7) | 2897(34.9) | <0.0001 | 58% of the data are missing. |
| **Prehospital Mechanical CPR, n (%)** | 248(0.5) | 0 | <0.0001 | 8 |
| **ED Mechanical CPR, n (%)** | 697(1.5) | 320(3.9) | <.0001 | 93% of the data are missing. |
| **Call to ED time (min)** | 44.5(2432.6) | 22.7(8.9) | <.0001 |  |

**ED, emergency department; ROSC, return of spontaneous circulation; CPR, cardiopulmonary resuscitation**
